# The General Nursing Council of England and Wales

**Published:** 1921-07-23

**Authors:** 


### THE GENERAL NURSING COUNCIL OF ENGLAND AND WALES.

The sixteenth meeting of the General Nursing Council of England and Wales, officially described as an emergency meeting, was held on Thursday, July 14, at the Ministry of Health. There were present Mr. J. C. Priestley, K.C. (Chairman), Dr. Bedford Pierce, Dr. Goodall, Miss Coulton, Miss Dowbiggin, Mrs. Bedford Fenwick, Miss MacCallum, Miss Macdonald, Miss Sparshott, Miss Lloyd Still, Miss Swiss, Miss Worsley, and the Registrar (Miss Riddell). Letters of apology for absence were received from Sir Jenner Verrall, Lady Hobhouse, and others. Miss Cox-Davies' serious illness prevented her from attending.

The Chairman read a letter from the General Nursing Council for Scotland, received on July 6, dealing with the draft rule relating to the removal from the English Register of nurses also registered in Scotland or Ireland, a subject which has already been before the English Council. The Scottish Council suggests that when a nurse's name is removed from one Register the Disciplinary and Penal Cases Committee should have power to accept the evidence already produced, and decline to proceed with frivolous appeals. The English Council holds that to agree to such an amendment of the rule would deprive a nurse on the English Register of her right of appeal in the English High Court. Mrs. Bedford Fenwick expressed the hope that the Council would adhere to its decision, and that it would not alter the rule, and so deprive English nurses of a second appeal to their own original Council if they desired to have it. She was supported by Miss Sparshott, and the Council agreed to adhere to its rule, and to advise the Scottish Council accordingly.

### What will Constitute a Training-School?

In reply to a request from the Society of Superintendents of Tuberculosis Institutions that the Council would receive a deputation on the question of the training given in tuberculosis institutions qualifying as part of general training, the Council decided that there is no object in receiving a deputation at present. The matter will be dealt with when the question of reciprocal training is under consideration. The Mildmay Mission Hospital has asked if it will be possible for a representative of the General Nursing Council to visit the hospital and discuss how best to bring it up to the required standard to qualify for registration as an approved institution. The hospital has fifty beds, and gives a three years' course. Dr. Goodall pointed out that the Council has not yet decided upon the size which shall qualify a hospital to be approved as a training-school; and Mrs. Bedford Fenwick added that the principles upon which approval would be granted have not been settled. In due course hospitals desiring to be approved will be inspected. It was agreed that a letter pointing out these facts should be sent.

### Minister Ready to Sign Rules.

The Chairman read a letter from the Minister of Health stating his readiness to sign at once the draft rules for the admission of existing nurses to the Register, subject to the omission for the time being of Rule 16, which rule provides for reciprocal registration with Ireland subject to a uniform standard of qualification. The Minister is engaged in negotiations with Scotland with the view to securing its adoption of a standard uniform with that of England and Ireland. A settlement is in sight, and he does not anticipate that the outstanding points will be incapable of a satisfactory solution.

Miss MacCallum, invited by the Chairman to make a statement, said that in view of one's experience of different Ministers she would like to be assured that the matter would only be in suspense for a short time, and that meantime Scottish nurses should not be allowed on the English Register by other means. The Chairman reminded her of Rule 4, which provides that an applicant is permitted to apply for admission to the Register of the General Nursing Council for England and Wales, notwithstanding she may have been trained in Scotland or Ireland. Miss MacCallum thereupon moved that the Council should agree with the Minister's letter. Mrs. Bedford Fenwick seconded, and described the letter of the Minister as most enlightened. The matter had been in abeyance for very long, and had held up the Register. To her mind there is no provision in any of the Acts for reciprocity. As the law officers of the Crown had ruled otherwise, the Registration Committee of the English Council drafted Rule 16, providing for a uniform standard of qualification, and applying only to Ireland, which had agreed to a uniform standard. She believes it to be a good thing for the English Council to put aside the question of reciprocity, and to open the Register at once as Sir Alfred Mond proposes. If such action would constitute any injustice to nurses trained in Scotland or Ireland she would oppose it, but it does not, for all nurses so trained can come on to the English Register on the same conditions as English trained nurses. The following resolution was then carried unanimously: "That the suggestion contained in the Minister's letter received on July 9 be adopted, with the consequence that Rule 16 of the printed rules be eliminated, at all events for the present." The rules were thereupon sent down for signature. Mrs. Bedford Fenwick proposed, and Miss Swiss seconded, that the Registration Committee should have power to consider applications for registration, and to submit a list of applicants recommended to the Council for its approval: also to report to the Council applications other than those recommended for approval. After some discussion on the method to be adopted of announcing to the public that the Register is opened, in which Dr. Bedford Pierce, the Chairman, Mrs. Bedford Fenwick, and Miss Dowbiggin took part, Dr. Bedford Pierce proposed "That the Council on receipt of the Minister's signature to the Rules shall announce that the Rules have been signed, and that the Register is opened, and that the circumstances should be explained to the public." It was further suggested that in addition to the British nursing papers the announcement should be made in the Colonial nursing papers and in *The Times* and *Daily Mail*.

The report of the Registration Committee was received, agreed, and adopted. It was suggested that the time for printing the first Register should be extended for six months. The opening of the Register had been held up for so long that it would be waste of money to print it next January as had been arranged. A General Purposes Committee to deal with domestic matters in connection with the headquarters of the Council at 12 York Gate was appointed, consisting of Mrs. Bedford Fenwick, Miss Cox-Davies, Miss Villiers, Miss Coulton, and Miss MacCallum. The first three have already acted as a Committee in connection with the furnishing and equipment of the premises.

### The Draft Syllabus.

The report of the Education and Examination Committee was presented by Miss Lloyd Still, Chairman. In reference to a letter received from the Irish General Nursing Council on the subject of the admission of nurses to the Register by examination, the report recommends closer co-operation with the other two Councils in this matter, a basis of agreement between the three countries with the view of having the same set of rules, and conference between members of the three Councils to lay down points to be agreed on before the rules are drafted. An interesting discussion took place on the recommendation that the draft syllabus of lectures and demonstrations for education and training should be adopted in its amended form and its publication sanctioned. In view of the opposition that has arisen in regard to the Syllabus, Miss Sparshott suggested that the Syllabus of education and training should be delayed or withheld until an examination syllabus is sent out. She wholly approves of the Syllabus, but people are not understanding it, and some of them apparently do not wish to understand it. The whole point is in the interpretation of it. Anyhow, it would be desirable to let the training schools know that an examination syllabus is coming. Miss Worsley supported this. She declares there is a great deal of feeling in Liverpool about the Syllabus, and perhaps an examination syllabus would help to allay the alarm. To her point that she would have liked the Syllabus arranged more on the lines of the Scottish one, the Chairman replied that the Scottish plan had been considered and set aside in favour of their own. After further discussion, Miss Sparshott moved an amendment that the Syllabus be held back until the syllabus for examination be ready to publish with it. This was seconded by Miss Worsley, but Miss Sparshott subsequently withdrew her amendment on it being pointed out that its effect would be to show that the Council is not prepared to adopt the Syllabus. The original motion was then put and carried unanimously. Dr. Bedford Pierce moved that it be a recommendation to the Education Committee that in issuing the Syllabus it should take into consideration the desirability of sending out some document dealing with the difficulties raised and pointing out that the Syllabus of training is not the Syllabus of examination. This was seconded by Miss Worsley and carried. The Education Committee further recommends the rescission, owing to the lapse of time, of a previous resolution of the Council as to voluntary State examinations. The effect of this rescission is that there will be no voluntary examination; the first State examination, which will be compulsory, will be held in July 1924. The Committee reports that the Draft Syllabus for training for children's and fever nurses is now under consideration.

The report of the Education and Examination Committee was received, agreed, and adopted.

Joint Deputation from the Poor Law.

At this stage the deputation appointed by the Conference held on July 8 (see The Hospital, July 16, page v) of representatives of Boards of Guardians having training-schools for nurses was received. It included also Mr. Chapman (Holborn), the Rev. W. Mahon (Wakefield), and Canon Glossop (St. Albans), all representatives of the Poor-law Unions Association. The deputation was introduced by the Rev. P. S. G. Propert, the President of that Association, who stressed the desire of the Conference to maintain a high standard of nurse-training. Poor-law authorities recognise that the adoption of the principle of registration, of which they heartily approve, involves the standardisation of training, qualification, and examination. The Syllabus of training should make it clear that the theoretical instruction and the elementary-science subjects are intended to be treated in a brief and elementary outline. If a syllabus of examination could be issued it would be a great advantage, and it should outline specifically the subjects on which the candidate will be examined. Further points Mr. Propert urged were that the first compulsory State examination should be deferred until July 1925, so as to give an opportunity to all training-schools to remodel themselves so as to meet the requirements of the Syllabus; and that among the examiners should be representatives of medical officers and superintendents and matrons of infirmaries. Mr. Percival elaborated Mr. Propert's remarks, and dwelt on the alarm of the smaller schools. The larger ones, he said, speaking generally, and subject to small amendments, have already made up their minds that there is nothing in the Syllabus they cannot accept. The alarm is largely due to the form rather than to the matter of the Syllabus, and he specified various alterations which the deputation suggested.

Miss Lloyd Still, at the request of the Chairman, replied to the points raised by the two previous speakers. The object of the Syllabus is to simplify matters as much as possible by giving the whole of the first year's work into the hands of the sister-tutors and teaching-sisters. Objection had been taken to the inclusion of elementary science, but under that come drainage and ventilation, and other matters very necessary for the nurse to be acquainted with, and in the second and third years it includes matters essential for the nurse who goes on to district work; it is therefore left optional in these two years. Every nurse should know something of gynæcology; she meets with gynæcological conditions in private work, and all nurses do not take midwifery. Other specific points raised by Mr. Percival were answered by Miss Lloyd Still. In reply to a question, the Chairman pointed out that a great amount of time has been taken up with the rules for existing and intermediate nurses, so that the rules for future nurses have not yet been considered. Mrs. Bedford Fenwick opined that in regard to examiners medical practitioners and nurses would be included, the one for theory, the other for practice.

Points concerning the training of male nurses, the question of amalgamation for training purposes of various institutions, and the establishment of preliminary courses were raised. The Chairman of the General Nursing Council promised consideration to all, and said he would welcome any suggestions or schemes which might be sent him. After thanks from Mr. Frater (Chairman, Tynemouth Board of Guardians) to the Council for their cordial reception, in which he was seconded by Mrs. Roberts (West Derby Union), and supported by the Rev. P. S. G. Propert, the deputation withdrew.

The report of the Finance Committee was then received and adopted, and the Council meeting, stated to be the last before the summer holidays, concluded.

